# Treatment initiation by positive liquid biopsy alone in primary central nervous system lymphoma: A retrospective analysis of a multi-institutional study

**DOI:** 10.1093/noajnl/vdaf274

**Published:** 2026-01-12

**Authors:** Masaki Mitobe, Satoshi Shibuma, Haruhiko Takahashi, Jotaro On, Toru Takino, Keita Kawabe, Yoshihiro Mouri, Shunsuke Kumagai, Takashi Kozakai, Akihito Momoi, Naomi Suzuki, Takao Fukushima, Takaharu Suzuki, Hiroyuki Kuroda, Etsuji Saji, Kimihiko Nakamura, Hideki Hashidate, Asa Nakahara, Takahiro Tomita, Jun Watanabe, Yoshihiro Tsukamoto, Masayasu Okada, Tetsuya Hiraishi, Shinya Yamashita, Takuya Akai, Satoshi Kuroda, Akiyoshi Kakita, Hirohito Sone, Jun Takizawa, Makoto Oishi, Manabu Natsumeda

**Affiliations:** Department of Hematology, Endocrinology and Metabolism, Faculty of Medicine, Niigata University, Niigata, Japan; Department of Neurosurgery, Brain Research Institute, Niigata University, Niigata, Japan; Department of Neurosurgery, Brain Research Institute, Niigata University, Niigata, Japan; Department of Neurosurgery, Brain Research Institute, Niigata University, Niigata, Japan; Department of Neurosurgery, Brain Research Institute, Niigata University, Niigata, Japan; Department of Neurosurgery, Brain Research Institute, Niigata University, Niigata, Japan; Department of Neurosurgery, Niigata Prefectural Central Hospital, Joetsu, Japan; Department of Neurosurgery, Niigata Prefectural Shibata Hospital, Shibata, Japan; Department of Neurosurgery, Niigata Prefectural Central Hospital, Joetsu, Japan; Department of Hematology, Niigata Prefectural Central Hospital, Joetsu, Japan; Department of Hematology, Niigata Prefectural Central Hospital, Joetsu, Japan; Department of Neurology, Niigata Prefectural Shibata Hospital, Shibata, Japan; Department of Neurology, Niigata Prefectural Shibata Hospital, Shibata, Japan; Department of Hematology, Endocrinology and Metabolism, Faculty of Medicine, Niigata University, Niigata, Japan; Department of Hematology, Niigata Prefectural Shibata Hospital, Shibata, Japan; Department of Hematology, Niigata Prefectural Shibata Hospital, Shibata, Japan; Department of Neurology, Niigata City General Hospital, Niigata, Japan; Department of Neurosurgery, Niigata City General Hospital, Niigata, Japan; Department of Pathology, Niigata City General Hospital, Niigata, Japan; Department of Pathology, Brain Research Institute, Niigata University, Niigata, Japan; Department of Neurosurgery, Graduate School of Medicine and Pharmaceutical Science, University of Toyama, Toyama, Japan; Department of Neurosurgery, Brain Research Institute, Niigata University, Niigata, Japan; Department of Neurosurgery, Brain Research Institute, Niigata University, Niigata, Japan; Department of Neurosurgery, Brain Research Institute, Niigata University, Niigata, Japan; Department of Neurosurgery, Brain Research Institute, Niigata University, Niigata, Japan; Department of Neurosurgery, Niigata Prefectural Central Hospital, Joetsu, Japan; Department of Neurosurgery, Graduate School of Medicine and Pharmaceutical Science, University of Toyama, Toyama, Japan; Department of Neurosurgery, Graduate School of Medicine and Pharmaceutical Science, University of Toyama, Toyama, Japan; Department of Pathology, Brain Research Institute, Niigata University, Niigata, Japan; Department of Hematology, Endocrinology and Metabolism, Faculty of Medicine, Niigata University, Niigata, Japan; Department of Hematology, Endocrinology and Metabolism, Faculty of Medicine, Niigata University, Niigata, Japan; Department of Neurosurgery, Brain Research Institute, Niigata University, Niigata, Japan; Department of Brain Tumor Biology, Brain Research Institute, Niigata University, Niigata, Japan; Advanced Treatment of Neurological Diseases Branch, Brain Research Institute, Niigata University, Niigata, Japan; Department of Neurosurgery, Brain Research Institute, Niigata University, Niigata, Japan; Department of Brain Tumor Biology, Brain Research Institute, Niigata University, Niigata, Japan; Advanced Treatment of Neurological Diseases Branch, Brain Research Institute, Niigata University, Niigata, Japan

**Keywords:** diagnosis, droplet digital PCR, liquid biopsy, *MYD88* L265P, primary central nervous system lymphoma

## Abstract

**Background:**

The gold standard for the diagnosis of primary central nervous system lymphoma (PCNSL) remains surgical biopsy, but it carries the risk of complications, and operability depends on the size and location of the lesion or the patient’s condition. Previously, we have reported the reliable detection of *MYD88* L265P-mutant droplets in cerebrospinal fluid (CSF) cell-free DNA (cfDNA) of PCNSL using droplet digital PCR (ddPCR). In the present study, we conducted a multi-institutional study to diagnose and initiate treatment for PCNSL by liquid biopsy alone without a surgical biopsy.

**Methods:**

We analyzed 10 patients from 5 institutions who were deemed difficult to biopsy surgically and were subsequently treated based on the detection of *MYD88* L265P-mutant droplets in their CSF cfDNA. CSF was obtained by lumbar puncture at each institution, cfDNA was extracted at Niigata University, and ddPCR was performed to detect *MYD88* L265P-mutant droplets.

**Results:**

Surgical biopsy was not performed in 8 patients because the lesions were located in deep locations, such as the brainstem, and in 2 patients because of low performance status and/or advanced age. *MYD88* L265P-mutant droplets were detected in all cases. Treatment response was observed in all cases. In a patient with chronic renal failure, liquid biopsy was helpful to rule out possible relapse.

**Conclusions:**

Liquid biopsy for the diagnosis of PCNSL has the potential to replace surgical biopsy with a less-invasive method and early diagnosis, leading to early treatment and possibly better outcomes. Further large-scale studies are warranted to establish the reliability of this approach.

Key Points

- *MYD88* L265P mutations of PCNSL can be detected in CSF cfDNA by droplet digital PCR.
- Treatment can be initiated without surgical biopsy by detecting the *MYD88* mutation in high-risk situations (elderly or frail patients, deep locations).

Importance of the Study

We have previously reported on the reliable detection of MYD88 L265P-mutant droplets in the cell-free DNA of cerebrospinal fluid from patients with primary central nervous system lymphoma (PCNSL) using droplet digital PCR. With this method, PCNSL can be diagnosed without a surgical biopsy, which depends on the size and location of the lesion or the patient’s condition. This approach allows us to initiate treatment rapidly and less invasively. The present study is a preliminary investigation aimed at determining the feasibility of a liquid biopsy-only approach for diagnosing PCNSL. In the future, as a liquid biopsy-based diagnostic method is established, it may be possible to avoid surgical biopsies in more cases, even in those that are not difficult to biopsy, and this could possibly lead to better outcomes.

The gold standard for the diagnosis of primary central nervous system lymphoma (PCNSL) remains surgical biopsy,^1^ but it carries the risk of worsening neurological symptoms due to hemorrhage, and depending on the size and location of the lesion or the age and general condition of the patient, surgical biopsy can be risky. We have previously reported the usefulness of liquid biopsy detecting *MYD88* L265P from cell-free DNA (cfDNA) in cerebrospinal fluid (CSF) for the diagnosis of PCNSL.^2^^,^^3^ In the first study, we found a 100% match in *MYD88* L265P status between tumor DNA and CSF cfDNA in 21 central nervous system lymphoma patients with paired samples.^2^ In this paper, the term “liquid biopsy” is defined as the analysis of cfDNA in CSF. *MYD88* L265P mutation is found in as high as 85% of PCNSL.^4^ Besides PCNSL, *MYD88* L265P mutations are found in various frequencies of B-cell type lymphomas, such as systemic diffuse large B-cell lymphoma (DLBCL) (29%) and lymphoplasmacytic lymphoma (LPL) (over 95%),^5^ but not in other primary or metastatic brain tumors. Therefore, when *MYD88* L265P mutations are found in CSF cfDNA in patients with a suspected brain tumor, a presumptive diagnosis of PCNSL can be reached if systemic DLBCL and LPL are ruled out. Here, we report 10 such difficult-to-biopsy or frail cases treated by positive liquid biopsy alone.

## Methods

At Niigata University and 4 participating facilities located within a 250 km radius in the Niigata and Toyama prefectures (Figure S1) during the study period of January 2017 to June 2025, 10 patients who were deemed difficult to surgically biopsy and subsequently treated based on the detection of *MYD88* L265P-mutant droplets from CSF cfDNA were analyzed retrospectively in the study. The infeasibility of surgical biopsy and treatment protocols was determined at the discretion of the attending physicians and through discussion with a neurosurgeon at Niigata University (M.N.). These cases included lesions that were adjacent to the pyramidal tract and/or near the brainstem, potentially causing symptoms such as hemiparesis, diplopia, medial longitudinal fasciculus syndrome, facial palsy, and hearing loss if hemorrhage were to occur during needle biopsy. This study was conducted after obtaining approval from the Institutional Review Board (IRB) at Niigata University Hospital (G2018-0008), and approval to conduct the study was obtained from all participating facilities. Written consent was obtained from patients and their families for liquid biopsy. This study was conducted solely in a research setting, although the IRB allowed the results to be returned to the attending physician and shared with the patient at the physician’s discretion. CSF was obtained by lumbar puncture when PCNSL was suspected on MR images. CSF from the participating facilities was collected in Streck Cell-Free DNA blood collection tubes (Streck tubes) (Streck, La Vista, NE, United States), which can stabilize cfDNA for up to 14 days at room temperature,^6^ and sent to the Department of Neurosurgery, Niigata University, for analysis. CSF was centrifuged at 1500*g* for 10 min to remove cells. CSF samples collected at Niigata University were immediately centrifuged under the same conditions and stored at −80 °C. Cell-free DNA was extracted using the Maxwell RSC ccfDNA Plasma Kit (RSC; Promega, Leiden, Netherlands) from 1.0 mL of CSF to 60 μL of elution buffer. Droplet digital PCR (ddPCR) was carried out with 9 μL of extracted cfDNA per well using the QX200 Droplet Digital PCR system and *MYD88* L265P primer probe mix (Bio-Rad, Hercules, CA). We considered cases with a variant allele frequency (VAF) of at least 0.1% and at least 3 detected mutant and/or wild-type droplets to be definite mutant cases. Conversely, we considered cases with a VAF of less than 0.1% or 2 or fewer mutant droplets, and 3 or more wild-type droplets, to be wild-type cases. Cases with only 2 or fewer mutant and wild-type droplets detected were considered undeterminable. Droplet digital PCR was performed in at least 2 separate wells for each case.^2^^,^^3^^,^^7^ In 10 cases in which *MYD88* L265P-mutant droplets were detected, and systemic lymphoma was ruled out, treatment was initiated based on the discretion of the physicians at the participating facility.

## Results

**Figure 1.**
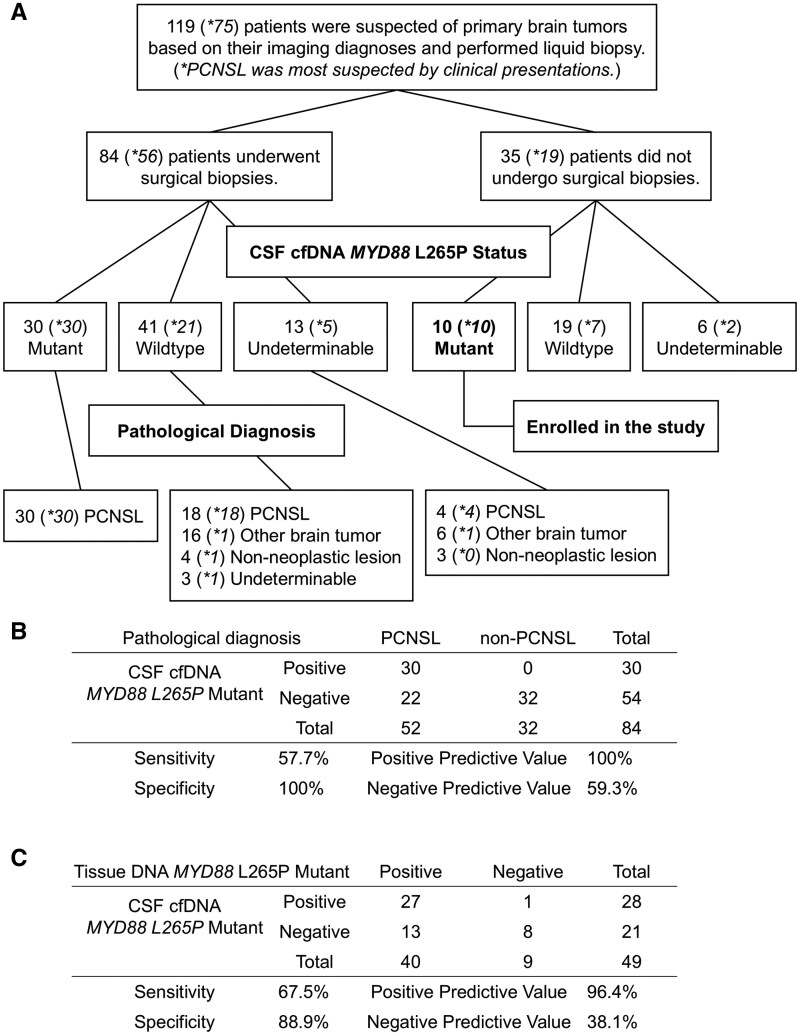
(A) Patient flow of the study. From January 2017 to June 2025, 119 patients who were suspected of having primary brain tumors underwent liquid biopsies. Of those patients, 75 were most suspected with PCNSL based on imaging and clinical findings. Ten patients who were diagnosed with PCNSL based on liquid biopsy alone and did not undergo surgical biopsy were enrolled in the study. (B) A 2 × 2 table showing the sensitivity and specificity of CSF MYD88 L265P detection for the diagnosis of PCNSL in the 84 patients who underwent a surgical biopsy. (C) A 2 × 2 table showing the sensitivity and specificity for detecting the MYD88 L265P mutation in CSF and biopsy tissue in patients diagnosed with PCNSL by surgical biopsy. Tissue DNA was extracted from frozen or formalin-fixed, paraffin-embedded tissue samples and analyzed using ddPCR. DNA from tissue was available in 49 out of 52 patients diagnosed with PCNSL. cfDNA, cell-free DNA; CSF, cerebrospinal fluid; ddPCR, droplet digital PCR; PCNSL, primary central nervous system lymphoma.

**Figure 2.**
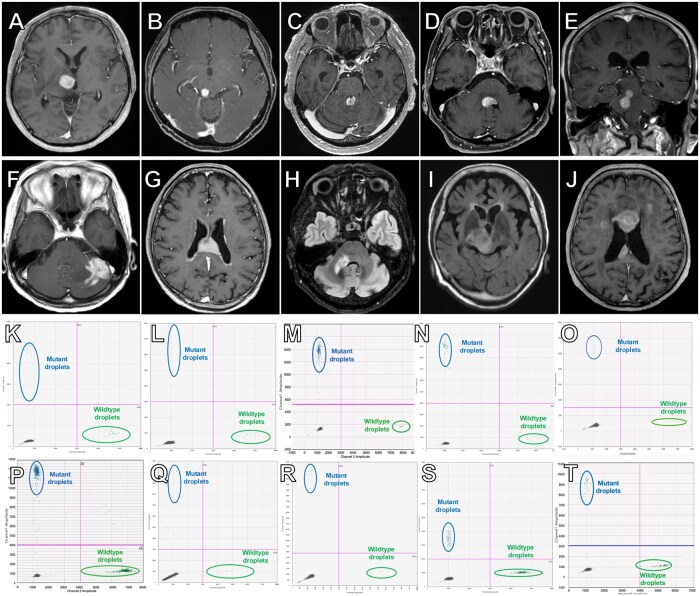
Head MR images of patients #1-10 are shown in (A-J), respectively. (A-G) and (J) are all post-contrast T1 images. (H) and (I) are FLAIR images, because post-contrast MR images could not be taken due to chronic renal failure. The ddPCR results of CSF from patients #1 to 10 are shown in (K-T), respectively. *MYD88* L265P-mutant droplets were detected in all cases. CSF, cerebrospinal fluid; ddPCR, droplet digital PCR.

Patient flow of the study is summarized in Figure 1A. In 84 patients with suspected primary brain tumors who underwent biopsy, the sensitivity and specificity of liquid biopsy for pathological diagnosis of PCNSL were 55.8% and 100%, respectively (Figure 1B). In 49 patients with a pathological diagnosis of PCNSL with known MYD88 L265P status, the sensitivity and specificity of liquid biopsy for the mutation were 67.5% and 88.9%, respectively (Figure 1C). Patient demographics of the 10 patients enrolled in the study, who underwent treatment for PCNSL with a positive liquid biopsy and no histopathological confirmation, are summarized in Table 1. The patients’ ages ranged from 60 to 87 years old, with a median age of 72 years. There were 4 men and 6 women. In 8 patients, surgical access was deemed to be difficult because the lesions were located in deep locations such as the brainstem. In 2 patients, the surgeon was hesitant to perform a surgical biopsy because of low performance status and/or advanced age. In 2 patients, post-contrast MR images could not be taken due to chronic renal failure. Systemic lymphoma, as well as pulmonary lesions related to tuberculosis and sarcoidosis, was ruled out by a negative whole-body CT in all cases. Cerebrospinal fluid was obtained by lumbar puncture in all patients. Corticosteroids were administered to patient #1 before liquid biopsy. Cerebrospinal fluid cytology was classified as class V in only 1 case, while it was classified as classes I to III in the other cases studied. *MYD88* L265P-mutant droplets were detected by liquid biopsy in all cases. CSF flow cytometry was not performed in any of the cases. Variant allele frequency ranged from 31.0% to 96.7%, with a median of 51.1%. The turnaround time from obtaining CSF and ddPCR results to come back ranged from 0 to 10 days, with a median of 2 days. The time to treatment initiation in patients treated only with a positive liquid biopsy was unchanged compared to the 30 patients who underwent surgical biopsy (median 12.5 days [IQR25-75: 9-15.75 days] vs median 11.5 days [IQR25-75: 6-13.75 days], *P* = .44, Mann-Whitney test). The percentage of patients whose CSF was sent from outside institutions was significantly higher in the positive liquid biopsy-only group (4/10 [40%] vs 2/52 [3.8%], *P* = 0.0047 Fisher’s exact test), potentially contributing to longer time to treatment in this group. The ddPCR results and MR images of all patients are shown in Figure 2. Along with radiographical findings, all cases were clinically diagnosed as PCSNL. Methotrexate-based chemotherapy was administered in 6 patients, tirabrutinib in 1 patient, 2 patients received whole brain radiation therapy (WBRT), and 1 patient received stereotactic radiosurgery using CyberKnife. All patients showed response to treatment. A complete response (CR) or unconfirmed complete response (CRu) was obtained in 4 patients, and a partial response (PR) in the remaining patients.

**Table 1.** Patient demographics of the 10 patients enrolled in the study

Patient No.		1	2	3	4	5	6	7	8	9	10
Age, sex		60, M	65, F	70, M	70, F	72, M	72, F	73, M	75, F	76, F	87, F
Tumor location		Rt thalamus, midbrain	Midbrain	Fouth ventricle	Brainstem	Pons	Cerebellum, Rt frontal lobe	Corpus callosum, periventricular	Rt middle cerebellar peduncle	Rt thalamus, midbrain	Splenium
Reasons for not performing a surgical biopsy		Deep location, steroids, low PS	Deep location	Deep location	Deep location	Deep location	Low PS	Deep location	Deep location, Low PS, renal failure	Deep location, renal failure	Low PS, age
CSF	Protein (mg/dL)	33	59	479	93	72	85	217	67	95	63
	β2-MG (mg/L)	2.38	5.2	5.84	N/A	4.7	N/A	9.63	2.86	3.77	N/A
	sIL-2R (U/mL)	N/A	N/A	N/A	N/A	N/A	704	1849	<90	798	422.1
	MNCs (/μL)	3	9	7	2	7	3	150	5	11	38
	PMNs (/μL)	0	0	0	1	6	0	0	0	0	<2
	Cytology	Class I	Class I	N/A	Class I	Class I	Class II	Class V	Class I	Class I	N/A
ddPCR (CSF)	cfDNA concentration (ng/μL)	18.3	4.7	1.2	12.1	4.7	23.2	32.5	1.8	8.1	7.7
	No. of mutant droplets	32	15	145	59	19	422	12	10	95	26
	No. of wild-type droplets	45	14	5	2	7	416	12	5	197	58
	VAF (%)	41.7	51.7	96.7	96.7	70.4	50.4	50.0	66.7	32.5	31.0
	*MYD88* status	Mutant	Mutant	Mutant	Mutant	Mutant	Mutant	Mutant	Mutant	Mutant	Mutant
CSF collection (day of admission^a^)		0	0	0	1	0	6	0	2	7	2
Turnaround time for ddPCR result (days)		2	2	0	2	9	10	1	3	2	4
Treatment		MPV	MPV	Tirabrutinib	R-MPV	MPV	R-MPV	MPV	WBRT	CK	WBRT
Treatment initiation (day of admission^a^)		6	14	3	16	9	21	15	9	30	11
Best response		PR	CR	PR	CR	PR	CR	PR	CRu	PR	PR
ECOG PS at liquid biopsy		3	1	3	1	2	3	2	3	2	3
ECOG PS at best response		3	1	1	1	2	1	1	3	2	3

Abbreviations: β2-MG, beta-2-microglobulin; cfDNA, cell-free DNA; CK, CyberKnife; CR, complete response; CRu, unconfirmed complete response; CSF, cerebrospinal fluid; ddPCR, droplet digital PCR; ECOG, Eastern Cooperative Oncology Group; MNCs, mononuclear cells; MPV, methotrexate/procarbazine/vincristine; N/A, not available; PMNs, polymorphonuclear neutrophils; PR, partial response; PS, performance status; R, rituximab; sIL-2R, soluble interleukin-2 receptor; VAF, variant allele frequency; WBRT, whole brain radiation therapy.

a Day 0 represents the day of admission.

**Figure 3.**
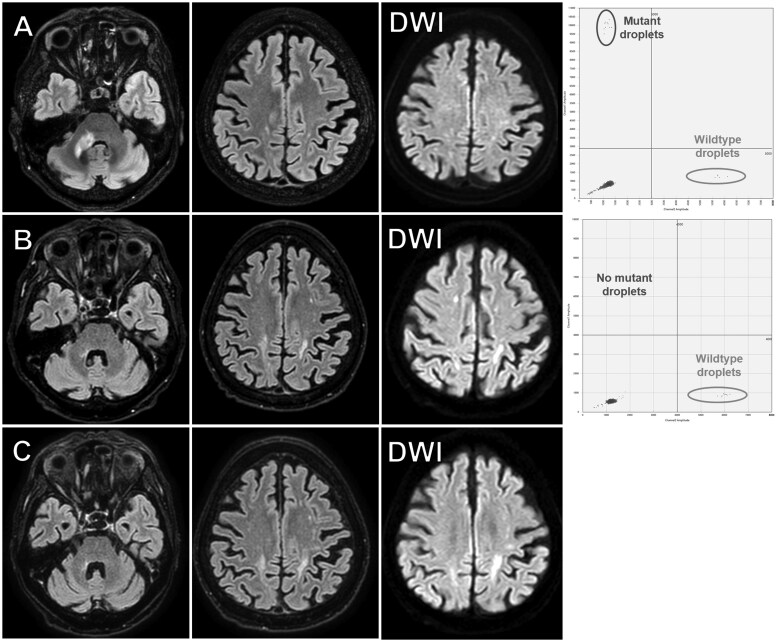
MR images (FLAIR and DWI) and the ddPCR result of patient #8 before treatment (A) and 5 months after treatment (B). New hyperintense lesions on FLAIR and DWI were observed in the bilateral parietal deep white matter but *MYD88* L265P-mutant droplets were not observed (B). MR images (FLAIR and DWI) 6 months after the initial observation of the parietal lesions (C). The lesions have primarily remained unchanged and recurrence of PCNSL was considered unlikely. ddPCR, droplet digital PCR; DWI, diffusion-weighted image; PCNSL, primary central nervous system lymphoma.

A repeat liquid biopsy was performed to confirm treatment response or minimal residual disease in 1 patient. Patient #8 presented with gait disturbance, vertigo, and diplopia. Due to a past history of chronic renal failure, gadolinium contrast was not used, but a FLAIR hyperintense lesion was found at the right middle cerebellar peduncle to dorsal pons (Figure 3A). Symptoms were progressive, and multiple *MYD88* L265P-mutant droplets were detected with a VAF of 66.7% by liquid biopsy (Figure 3A, right panel). Almost complete disappearance of the lesion was observed after WBRT. However, almost 5 months after the completion of WBRT, new hyperintense lesions on FLAIR and diffusion-weighted images were observed in the bilateral parietal deep white matter (Figure 3B). Ischemic lesions or recurrence were suspected; however, since the lesions were asymptomatic and a repeat liquid biopsy revealed no mutant droplets ­(Figure 3B, right panel), we chose to closely monitor the lesions by repeat MRI. Subsequent MR images have been taken bi-monthly, but the lesions have primarily remained unchanged 6 months after the initial observation of the parietal lesions (Figure 3C).

## Discussion

We reported a series of 10 cases in which treatment was commenced after the detection of *MYD88* L265P-mutant droplets from CSF cfDNA. Treatment response was observed in all patients, although histopathological confirmation was not obtained. For cases such as these, where surgical biopsy is difficult, liquid biopsy is a reasonable alternative for diagnosing PCNSL. A recent paper reviewing the advances in liquid biopsy for PCNSL suggested liquid biopsy as an alternative for the diagnosis of PCNSL in high-risk situations (ie, elderly or frail patients, deep locations).^8^

On the other hand, of the 10 cases, CR or CRu was achieved in only 4 cases after initial treatment. In many of these cases in which surgical biopsy was not performed, the patients were of advanced age, had chronic renal failure and/or low performance status, and therefore intensive chemotherapy could not be administered.

Furthermore, adding the CD20 monoclonal antibody rituximab to chemotherapeutic regimens is known to increase survival in PCNSL.^9^^,^^10^ However, since histopathological confirmation of CD20 positivity is not possible solely by the detection of *MYD88* L265P mutation in CSF cfDNA, the use of rituximab is not covered by health insurance in Japan. Rituximab was administered in only 2 out of 10 patients, which may have affected the low CR rate. Most lymphomas with *MYD88* L265P mutation are B-cell lymphomas,^11^ so CD20 can be postulated to be expressed in PCNSL cases in which *MYD*88 L265P-mutant droplets are detected from CSF cfDNA. Therefore, the use of rituximab in such cases is ideal. In the future, it may be possible to indirectly demonstrate CD20 expression using novel methods, such as cfDNA fragmentomics.

Merits of diagnosing PCNSL by liquid biopsy alone include avoiding invasive surgical biopsy procedures and shortening the time to treatment by bypassing biopsies in the operating room. Also, in the present study, we found that the diagnosis of patients is possible in distant facilities by sending CSF in Streck tubes, resulting in the safe treatment of difficult-to-diagnose PCNSL at local institutions. Surgical biopsy, even less-invasive stereotactic biopsy, can carry a morbidity rate of 5.8% for hematoma and 15.1% for cerebral edema, and a mortality rate of 2.8% has been reported.^12^ Complications in surgery or sampling error can result in a delay in the commencement of chemotherapy. Primary central nervous system lymphomas are fast-growing tumors, and oftentimes, the emergent use of corticosteroids is necessary before a biopsy can be performed. The use of corticosteroids before surgical biopsy or liquid biopsy can hinder their diagnostic accuracy.^13^^,^^14^ A lumbar puncture can easily be performed, and CSF specimens can be frozen after centrifugation, thereby eliminating time constraints. In this study, the period from admission to treatment initiation was unchanged in patients treated with a positive liquid biopsy alone, compared to CSF MYD88 L265-positive patients who underwent biopsy (median 12.5 days vs median 11.5 days, *P* = .44, Mann-Whitney *U*-test; Table S1); however, this could be shortened if this method alone could be considered definitive for diagnosing PCNSL. Liquid biopsy can be a reliable alternative in facilities where neurosurgical procedures are not readily available. In these circumstances, a liquid biopsy may be performed for cases in which a surgical biopsy is feasible.

One potential pitfall is the presence of PCNSL cases with wildtype *MYD88* or non-hotspot (L265P),^2^^,^^4^^,^^15^ reported in 15%-25% of PCNSL. In the surgical biopsy cohort, 9 out of 49 patients (18%) analyzed using surgical biopsy specimens were MYD88 L265 wild-type. Also, the sensitivity of CSF cfDNA to detect *MYD88* mutations was suboptimal at 67.5%, with the 13 false-negative cases (CSF negative, biopsy specimen positive) attributed to insufficient supply of CSF cfDNA, including improper handling and storage of CSF cfDNA (Figure 1C). Detection of *CD79B* mutations can also be diagnostic for PCNSL. However, 6 different amino acid substitutions have been observed at the hotspot *CD79B* Y196 codon, as well as many other sites.^15^ Other recurrent genetic alterations in PCNSL, such as *PIM1*, are also found across multiple sites^16^ and are more suited for detection by next-generation sequencing panels compared to ddPCR techniques. Next-generation sequencing panel testing for CSF cfDNA has been reported,^17^ but is still costly and time-consuming compared to ddPCR.

Another potential pitfall is that B-cell lymphomas other than PCNSL can also have *MYD88* L265P mutation. The *MYD88* L265P mutation is found in 29% of systemic DLBCL and over 95% of LPL and has been reported less frequently in Burkitt lymphoma, marginal zone lymphoma, and mantle cell lymphoma.^5^^,^^18^ These lymphomas can cause CNS involvement.^19^ In particular, rare LPL with CNS involvement, known as Bing-Neel syndrome (BNS), has a high positive rate of *MYD88* L265P and is critical to distinguish from PCNSL.^20^ DLBCL histologically transformed from LPL similarly has a high frequency of CNS involvement and *MYD88* L265P mutations,^21^ so a surgical biopsy is ultimately necessary to confirm the diagnosis. In fact, during the study period, there was a case in which *MYD88* L265P mutation was confirmed by liquid biopsy, though BNS was strongly suspected rather than PCNSL (Figure S2). Although there is no clinical picture or specific symptoms that can prove or exclude BNS,^20^ a prolonged clinical course, multiple cranial neuropathies, and elevated IgM levels in this patient were supportive of the diagnosis of BNS rather than PCNSL. However, a bone marrow examination was not performed. ^18^F-fluorodeoxyglucose (^18^F-FDG) PET/CT may also be useful to rule out secondary CNS lymphoma. When diagnosing PCNSL with this technique, exclusion by ^18^F-FDG PET/CT or bone marrow examination is important, especially if the imaging is atypical for PCNSL or immunoglobulin abnormalities are present. Another theoretical possibility is that clonal hematopoiesis involving the *MYD88* mutation could result in false positives, but the frequency is considered to be very rare and negligible.^22^

Of the 49 patients with a pathological diagnosis of PCNSL and known *MYD88* L265P status, 1 patient had a false-positive result for *MYD88* detection in the CSF cfDNA (Figure 1C). This patient had been receiving long-term steroid treatment, which could have affected this result. We have previously shown that short-term corticosteroid treatment before liquid biopsy can hinder the detection of mutant as well as wild-type droplets.^13^ However, it remains to be seen how long-term corticosteroid treatment can impact MYD88 L265P detection in tissue DNA and CSF cfDNA.

The potential application of liquid biopsy for determining treatment response, minimal residual disease, and relapse of systemic DLBCL and PCNSL is also highly anticipated.^2^^,^^8^^,^^23^ In 1 patient with renal failure in the present series, possible relapse was not apparent by a repeated liquid biopsy (patient #8, Figure 3B). In cases such as this, where the initial liquid biopsy is positive, serial monitoring is not only possible but also helpful in determining the treatment strategy.

Several limitations exist in the present study. This is a retrospective analysis of a multi-institutional study on liquid biopsy for brain tumors, including PCNSL. Therefore, diagnostic criteria and treatment protocols were not specified. Some important diagnostic methods, such as PET/CT and CSF flow cytometry, were not performed due to the difficulty of performing these diagnostic tests in Japan (PET/CT is restricted to patients in an outpatient setting and therefore not routinely performed in PCNSL patients in Japan who are usually emergently admitted for surgical biopsy and treatment) or unfamiliarity of these tests within the neurosurgical community. Radiation treatment was performed in some patients due to advanced age, renal dysfunction, and frailty, so the confirmation of a PCNSL diagnosis based on treatment response is suspect in some patients, as response after radiation can be observed in other brain tumor types as well.

The gold standard for the diagnosis of PCNSL remains surgical biopsy. However, efforts to diagnose central nervous system lymphoma by analyzing CSF DNA, including validation of laboratory-developed tests for clinical use, are increasingly being explored.^24^^,^^25^ With the spread of liquid biopsy, it has the potential to replace surgical biopsy with a less invasive method and early diagnosis, leading to early treatment and possibly better outcomes. The present study was a preliminary 1 determining the feasibility of a liquid biopsy-only approach to diagnosing PCNSL and was restricted to patients in which surgical biopsy was considered potentially harmful. In the future, as a liquid biopsy-based diagnostic method is established, it may be possible to avoid surgical biopsies in more cases, even those that are not difficult to biopsy. Further large-scale studies are warranted to establish the reliability of this approach.

## Supplementary Material

vdaf274_Supplementary_Data

## Data Availability

The data that support the findings of this study are available from the corresponding author upon reasonable request.
