# Supplementary material for: Treatment initiation by positive liquid biopsy alone in primary central nervous system lymphoma: A retrospective analysis of a multi-institutional study
Source: Neurooncol Adv. 2026 Jan 12;8(1):vdaf274. doi: 10.1093/noajnl/vdaf274 (PMC12978307; doi:10.1093/noajnl/vdaf274)
Supplement: vdaf274_Supplementary_Data [file vdaf274_supplementary_data.zip › Supplementary Table and Figure Legends..docx]

**Supplementary Table S1.** Turnaround time for CSF cfDNA MYD88 L265P mutant patients.

**Supplementary Figure S1.** The location of Niigata University (star) and four participating facilities (circle). They are located within a 250 km radius in the Niigata and Toyama prefectures, in northwest Japan. CSF samples were collected in Streck tubes at each facility and sent to Niigata University for analysis.

**Supplementary Figure S2.** This is a case of a 69-year-old woman who was suspected to have Bing-Neel syndrome rather than PCNSL. She had a history of unexplained sensorineural hearing loss for two years and experienced left retroorbital pain and diplopia one month prior to admission to the affiliated hospital. Post-contrast head MR images revealed contrast enhancement and swelling of multiple cranial nerves, the optic chiasm, and the ventral medulla oblongata (A). The ddPCR of the CSF revealed *MYD88* L265P-mutant droplets with a VAF of 55.6% (B). PCNSL was initially suspected. However, due to the patient's atypical imaging findings and prolonged clinical course, an elevated IgM level of 330 mg/dL, along with serum immunofixation electrophoresis was suspicious for an IgM kappa monoclonal protein at presentation. Bing-Neel syndrome was retrospectively suspected. A bone marrow examination could not be performed.
