# Supplementary figures and images for: Treatment initiation by positive liquid biopsy alone in primary central nervous system lymphoma: A retrospective analysis of a multi-institutional study
Source: Neurooncol Adv. 2026 Jan 12;8(1):vdaf274. doi: 10.1093/noajnl/vdaf274 (PMC12978307; doi:10.1093/noajnl/vdaf274)

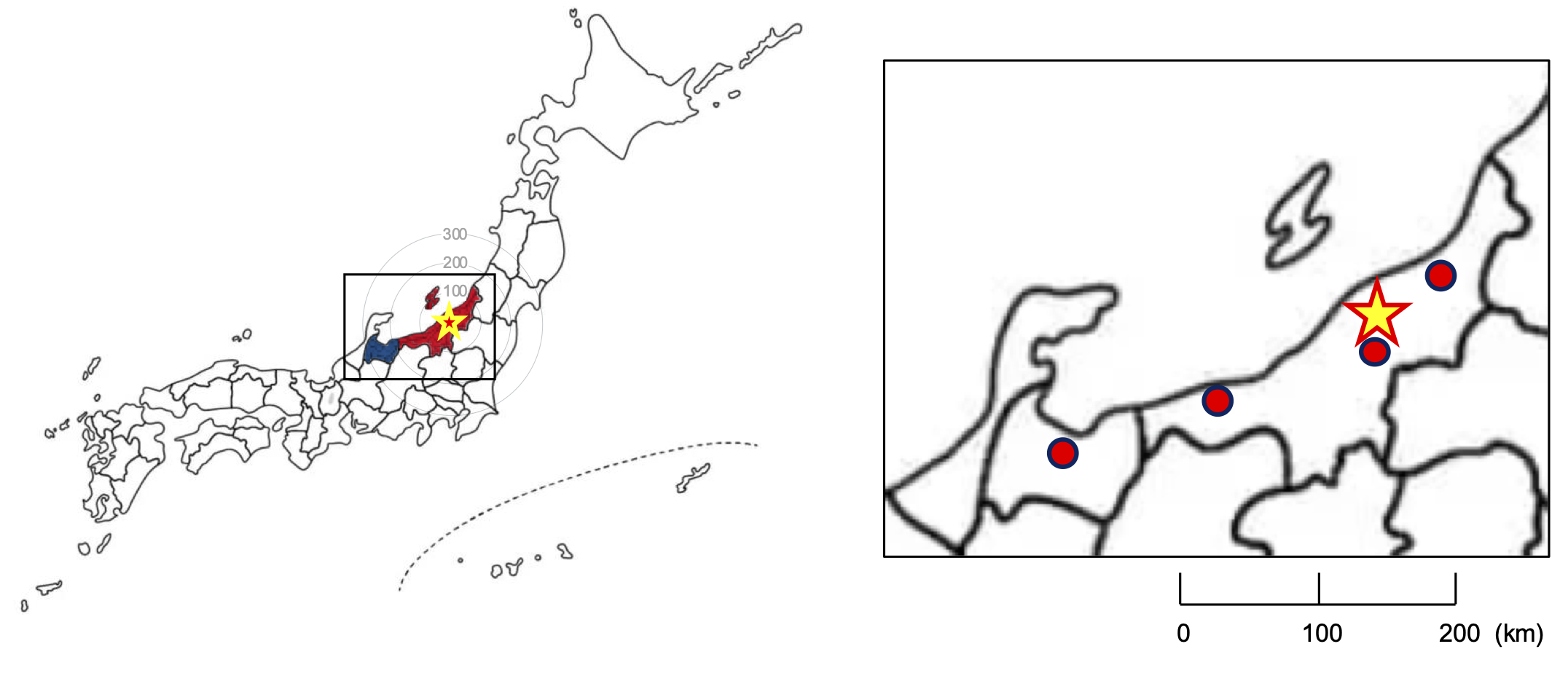

Supplement: vdaf274_Supplementary_Data [file vdaf274_supplementary_data.zip › Fig S1.tif]

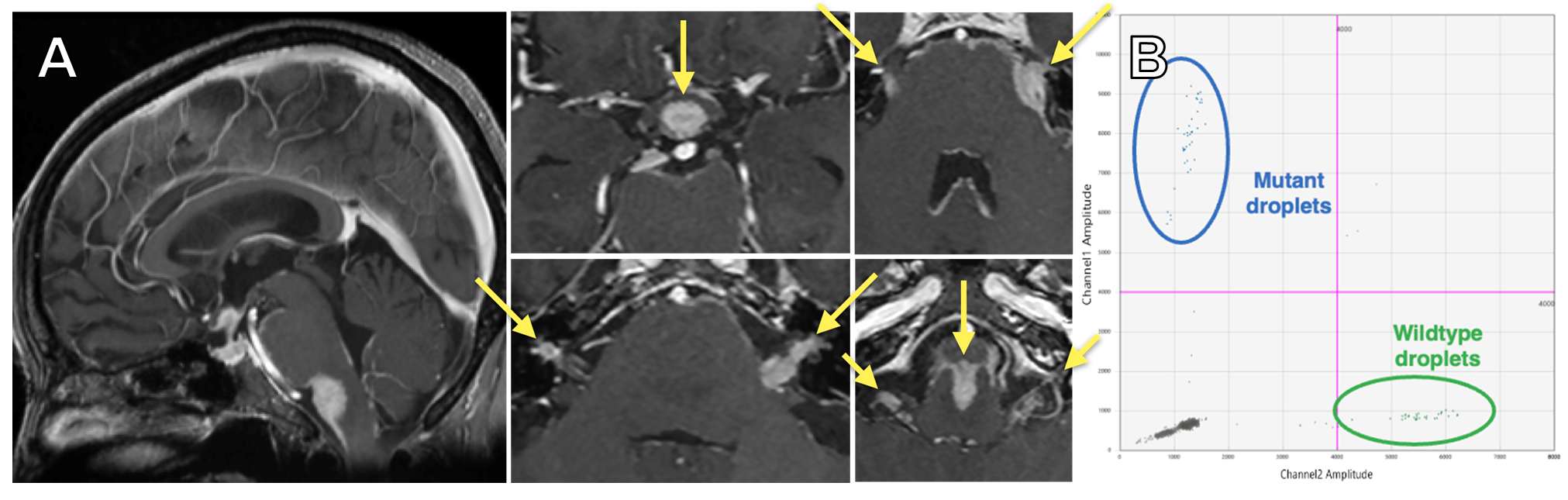

Supplement: vdaf274_Supplementary_Data [file vdaf274_supplementary_data.zip › Supplementary Figure 2_color_20250717.tif]
